# Dr. Bellamy, in Answer to Mr. Chalmers

**Published:** 1807-01

**Authors:** G. Bellamy

**Affiliations:** Plymouth


### *To the Editors of the Medical and Phyfical Journal.*

Gentlemen,

ALTHOUGH I think the best answer to the various observations of my opponents, Messrs. Chalmers and Dawson, will be found in the second part of my Case, which I hope to see in your next Number, as it is more than two months since it was delivered at your office; yet I am particularly called on to note the language, and illiberal conclusions of Mr. C. which no doubt he would fain dignify with the name of criticism. Had he confined his unjust insinuations to me, I should have passed them over with the contempt they deserve; but not satisfied with shewing his importance, and superior talents at my cost, he insidiously involves the Surgeons of the Royal Navy in general, under his presumptuous censure. Such censure does little credit to his head or his heart. Criticism is a talent of true learning, but scurrility and invective are propensities of a little mind, and generally arise from disappointment or envy. Mr. C. has excelled in that species of writing, which, unfortunately, is too justly complained of in medical authors; by which the world learns nothing, the profession is injured, and the writer exposed. I have for many years impatiently seen this kind of paper war, and have always promised myself to avoid such contests; and in reply, only to adhere to self-defence, and stubborn facts: and nothing short of the latter part of Mr. C's paper should have induced me to depart from my plan, or to trespass on the Medical Public, thus far to defend my brother officers, by exposing the absurdity, harshness, and unhappy disposition of Mr. C. I shall be told, perhaps, that I invited criticism, and therefore should expect it. I did so; it was on that ground chiefly I wrote. I want information; I have said so; where then is my harsh language, who have dared simply to narrate the truth, not to be a Case-maker, and blazon forth wonderful success? but have ventured to be among the few who acknowledge their disappointment, and that the wisest have yet more to learn than they have acquired.

After saying a few words to Mr. Dawson, I shall proceed to notice, in order, the suggestions and questions of both my premature critics, who have not done me the common justice to hear me out, and who thereby, I presume, shew themselves very deficient in the rules of criticism, and have only ultimately strengthened my opinion. It is not unworthy of remark, that Mr. C. comments no further than Jan. 30; and Mr. D. "waits not to hear the conclusion of this unfortunate case;" in which, if I had been successful, not a word would have been said. But as I am still before the public, I am not without hopes of learning something for which I wrote; for it cannot be supposed that I intended to instruct them, but merely to open a field of physiological and pathological discussion. I even think something may be acquired from Mr. D. because he writes with moderation, as a man of study, and like a gentleman.

I hope, from being so conspicuously brought into notice, that you will do me the favour to give this Paper an early insertion: the world must expect it; and I owe at least an attempt to defend my brethren, however far short I may fall of establishing my own ideas, or of having them them corrected and enlarged by ingenuous discussion. By absence at sea, I did not procure the Numbers, containing my Case, and the observations thereon, till late in this month. I am, &c.

*Plymouth, Oct.* 28, 1806. G. BELLAMY, M. D.

---

Mr. C. complains of a want of accurate anatomical description of the seat of the disease. I am at a loss to know what more could be said that is necessary, of the part affected; the space concerned is so confined, and the parts so simple, that minute delineation would only confound what must be readily understood. As to previous history, &c. the expression is so truly unintelligible, that it cannot be comprehended; and I have given all on that head which I thought necessary. If Mr. C. thinks otherwise, I may add, that the patient was known to me near two years before, and had only once complained of the same affection, which, as I have stated, was referred to a venereal cause; and he had all that time been troubled with a sense of narrowness in the urethra; but without seeking, so far as I know, any medical aid, or coming to me till about six months before, as I have stated. The seat and nature of the swelling was then the same as when he now applied; and as I was then so successful in removing the enlargement by mercurial friction on and around it; consequently I was not only justified, but authorized, to proceed on the same plan, the principle of promoting absorption by the excitement of mercurial friction, being confirmed by the former event, recommended by the highest authorities, and particularly enjoined (not exclusively by mercury, I allow, but I know of no means so powerful) where parts are concerned, the situation of whose extremity involves all the evil of uncertain cure from ulcer, sinus, fistula, filth, and deformity.

That man, surely, cannot be in his senses, who in the early stage of such affections, would act on any other principle but that of promoting absorption; and so say all the authors I have ever read, or teachers whom I have heard; and if they said otherwise, I should persevere not only to exert my own ideas (especially when supported by ample experience) in the outset, but to carry them on to the utmost; with difficulty relinquishing what I have known so often succeed, even after the full formation of matter, in much greater quantity, and where the cuticle seemed to be the only remaining parietes. But let it be remembered, bered, this perseverance is proportioned to the evils to be apprehended from a wound taking place. In the mean time, I deny that failing in my object, the destruction of the part, or suffering of the patient, will be increased; so far from it, I am firmly of opinion that the extent of an evil, which cannot be altogether avoided, is thereby lessened, by arresting the progress of disorganization, and promoting a partial absorption, by exciting the absorbents, and unburthening the blood vessels at a little distance from the apex.

On the 22d, I supposed it to be a chronic, &c. but in Mr. C's short and strange periods we are left to imagine whether he thinks it chronic or acute. Johnson will shew him various applications of the word SUPPOSE. Mr. C. assumes that I was doubtful; but the previous history, the present state, "no redness or heat, or sign of inflammation," explain what I thought, or believed, if Mr. C. likes the word better; and therefore I employed means which are not inapplicable even in inflammation, but are more expressly indicated in chronic affection. Have my adversaries yet to learn, that the same medicine may act in two very opposite ways; that, according to circumstances, dose, &c. opium and mercury will be either stimulant or sedative; that these are but relative terms; that stimulus will remove indolent tumours; and also, by a new and more powerful action, relieve inflammation, even locally?

On the 23d, the Swelling undoubtedly larger, &c. Now the word swelling is a comprehensive term, used expressly because I was between hope and fear of its chronic or acute nature, and beginning to apprehend the latter; but M. C. in the poverty of his cause, as all Quibblers do, works in words which are not mine; for on that day I have not used the word chronic; nor on the preceding, "hardly perceptible to the eye." And could I begin to apprehend fistula so soon, and not be aware of the approach of inflammation? I begin to think I am speaking to a person who has read or been taught little of the causes and nature of fistula, and seen still less of that dire disease.

As to the 24th, give me leave to tell Mr. C. that inflammation cannot exist without pain, (and I have said there was great pain) but most assuredly it did without reaction of the circulation, for he looked and felt weak; &c. not in remission, but tota die; and it often does so. Pray, has Mr. C. seen any thing of the yellow fever, whose essence, in the opinion of the most successful practitioners, is proved to be the most concentrated state of general general inflammation? Yet commonly, the patient is knocked down at once into a state, which the unwary would assert to be typhus, and begin, though not continue long, to cordialise. Ask Doctor Rush. Go to the West Indies, and see how much blood you may take from such a patient, and how often you may repeat v. s. in the first thirty hours, and what indications the pulse, skin, and countenance will give you; and see how certainly the fine spun deductions of systematic medicine will be found to lead to destruction; and what conviction will rush on the mind, on beginning to learn that we know little or nothing but by thinking and acting for ourselves, if we be vigilant in tracing the steps of Nature, and collate our observations with facts. The aridity of Mr. C's mind is great indeed, if he knows not that fomenting a painful swelling, with thick covering, will procure ease, will, by relaxing and expanding the vessels of such parts, relieve their surcharge, and greatly assist the introduction of ung. hydr.; the more desirable now, as inflammation being admitted, and the pain great, the act of rubbing should be diminished.

What is meant by the apostrophe in the Note of 25, is left to the learned. I have said, the patient had no rigors. Mr. C. has thought proper to add, that I seemed surprised. I might have said so, and I will say it now, for I never saw matter to any considerable amount formed without it, especially in a part so highly irritable; and next to the touch, what is the pathognomonic sign of formation of matter?

26th, I express my surprise at absence of rigor, altho' he has considerable fever. Here, for the correct reading of this day's quotation, the reader is requested to put a comma after the word *great,* which belongs to fever, and not to heat, as Mr. C. has written it. "What constitutes fever?" is indeed an important question. Had my critics waited a little, they would have spared so many unanswered interrogatories, and seen that my surprise is greater than their own. Is Mr. C. that imprudent man to himself and patient too, to disturb their minds with useless fears of danger or distress; generally speaking, when this duty is indicated, ours is nearly done. Mr. C. has also, in his bright conception, put cause for effect. Now, the want of explanation was the purposed consequence of his ease and confidence. The work of suppuration is now complete; that is, there is quite fever enough, sufficiently rapid progress of the phlegmon, to answer every purpose of an abscess; therefore more is not only unnecessary, but would be be injurious. What are the excesses of inflammation?— Here then I begin to wait upon Nature, or rather check her efforts in a strong plethoric habit; keep the patient *rather low*, quiet, clean, &c.

Be it known to Mr. C. that although the Navy has lost *his* excellence, it has nothing to seek which assiduity, zeal, minute and frequent visits to the sick, can give. If he were in the habit of writing Journals, he would know that the present is not detailed because it is employed in action, and that the series is the same, if at a regular daily hour we recapitulated. In one place he tells me I present a mass, and implies that my minutiæ are tedious. Here I am accused of silence, where nothing could be said, nor any thing done, otherwise than was done. The friction was discontinued after the 26th, morning, and not, as he would imply, the 27th. The remarks of the 28th being chiefly digressional, and employed in the exercise of reasoning, are passed over in silence, because Mr. C. does not exercise that talent.

The 29th is dignified with two apostrophes upon a simple act, to which no importance is attached by me, except its use. My diction may be very obscure, and Mr. C's comprehension may be dull; I acknowledge but one error, because I see no other in the 29th, and that is typographical, where the adverb *off* is used instead of the preposition *of*, in speaking of cutting the rapha perinæi.

On the 30th, Mr. C. winds up his Critique, without suggesting one single proposal of his own, which can tend to professional improvement or the good of Society. I cannot perceive in this day, that I have said the patient's health was good; "but a good strong habit, and promising state of the parts, are by no means incompatible with debility and giddiness from fever,. pain, and confinement. The bark was begun early in the morning of the 28th.— It has been a tedious but not painful task for me thus to overturn many erroneous assertions of Mr. C.; I shall proceed with more pleasure to those of Mr. D.

To Mr. Dawson I have first to make my acknowledgment for the neat paragraph with which he prefaces his remarks; it shews the man of sense, candour, and refined sentiment, earnest in the pursuit of truth, whilst it also exhibits the active character of him who greedily and gratefully feels it no task to throw his fire-brand, and presumes to tell you he means to injure no one. It is no trifling satisfaction thus to see the battle fought for me on the same page. The readers of the Medical Journal will the more more readily compare the different dispositions, and extent of capacity, of my adversaries; and, as well as myself, I doubt not, see how differently they have attacked, and each borne on points which the other has generally omitted. Whence arises *this* incongruity? It encourages me to expect seeing a third commentator, who may view through a different medium, and who, not improbably, may approach nearer the true focus; especially as by waiting till all the apparatus is before him, the more just refraction of the rays, shall paint the picture in its true colours on the retina of his mind. I have invited criticism on the phenomena of disease, and on the modus operandi of means, and have expressly solicited forbearance on the manner of description; every man has a peculiar way of expression, of course he must be allowed it; as to my practice it is not singular, though it may not be common; many of my reflexions on most subjects, I am not so bigotted as to be insensible of their outrēness, yet I am persuaded that they proceed from the labor of attention, and usually from the book of Nature. All this is not only compatible, permissible, but also necessary, and even highly useful in all liberal arts and sciences; and I shall ever proceed so to think, speak, and act for myself; and in so doing, ultimately for the good of the world in general, I hope. These principles may be restrained among pretenders, but are the indisputable right and duty of regularly educated men to exercise. So that all Mr. D. can consistently urge on this subject, is a difference of opinion, and not assume the arbitration of a judge. As to the value and pertinence of his comments, they are before the profession to estimate; and as to refutation, I think, after I am heard out, there will be none required. I can view all his remarks with complacency, a few only excepted, to be observed by and by. It is certain the patient had had *stricture*, confining the import of the word to narrowness of the urethra, shewn by some difficulty to begin micturition, and by the smallness of the stream for many years; as to neglect, it could not even lay at the patient's door, so little inconvenience did he usually suffer, that interference of bougies, &c. would only have been an officious meddling, had he ever complained of it, independent of the swelling; which he never did. I still say there was no sign of inflammation; that is, although pain, swelling, &c. &c. as quoted, may attend inflammation, yet as there was neither redness nor heat, increased vascularity, or excitement of the circulation, either of the part or system, so there did not not exist inflammation. All the symptoms I have enumerated (let it be observed that I am replying to the paragraphs in the order as they stand) exist in schirrous testes, and fifty other affections, not connected with inflammation. As to the event, judging by consequences is not fair argument, as my account is not a retrospective narrative after the event, but a daily account; so we must think and argue from day to day. "Why chronic?" Because not acute. "Is schirrous breast, and diseased prostrate gland, &c. designated by the quotation?" certainly yes. The affection was believed the consequence of venereal disease; and, if not, mercury is the best remedy, both as alterative and stimulant, in perhaps all chronic enlargements, schirrous excepted, therefore mercury was indicated. So that here Mr. D. has exceded his credentials, and his outset of modesty; and as to the use of five grains, bis de die. Answer, What time is required to rub it in, on a small space, or a part that was painful, but not inflamed?

Mr. D. speaks of increasing the inflammation, before I have allowed its existence; and attaches to its power the consequences which I deny were so produced; and which in my opinion, were even retarded thereby. I am persuaded, that in the view of the case, then entertained from day to day, that sedatives were not indicated, as there was neither heat, redness, nor re-action; and if there was a fear of fistula, I declare that practice to be reprobate, which should not be directed to obviate it in every possible way; little as Mr. D. seems to think of abscesses in perinæo, and the dreadful train of evils usually induced; therefore cataplasms were not resorted to, because they usually promote suppuration; and I still contend that the precise period was observed in the application, and change of means, according to the indications. But there are too many of our profession who prescribe without seeing the patient, and give a verdict before they have heard the evidence.

Mr. D's practice must have been very confined in the treatment of abscesses. He has never seen, it would appear, what I have witnessed a hundred times; large tumors full of matter, the effect of inflation. Buboes, for example, completely removed by absorption of the purulent, and other contents, into the general circulation, even after the integuments threatened to burst every minute; and by what means? By mercurial friction (not in this very advanced state upon its apex) on the borders of the tumour, and course of the lymphatics; relaxing the surface, face, and giving ease by fomentations at the same time. What misery is hereby cut short? Ulceration, filth, deformity, &c. Apply this to the importance and nature of the perinæum, and what it defends, and the question must be at rest.

Here let me caution the ignorant and indolent against the doctrine of Mr. D. who all along insisting upon inflammation, and condemning me for increasing it, now contradicts himself, and all authority, by advising the repeated introduction of bougies in an inflamed urethra; in vain would be the application of his antiphlogistic measures, should he persist, however cautiously, to dilate the passage while inflamed. Subdue the inflammation, and then dilate the urethra; but, in this case, even that would be only courting mischief, the patient never so much as being afflicted with stoppage of water; the greatest evil for many years being only a narrow flow of it. The least exciting cause of inflammation to a person with such a long standing affection is to be avoided. To talk of bougies, and cure, and that to a man who to live must bustle about, is mere theory. Let him follow lessons of temperance, and his life may be preserved for years; but now that a tumour is formed near the stricture, the passing of urine was not more difficult, and yet, though on the 24th, inflammation is admitted, *a bougie is to be introduced!* As the tumour was proved to be of an inflammatory nature, the question, simply, shall we promote suppuration, and most probably fistula, or endeavour to repel? The latter, I presume. But here I and Mr. D. are at variance in the means; and moreover, he is at variance with himself. After quoting the paleness and weakness of the patient, he accuses me for repelling, and he has just before advised debilitating means! Differing so widely in our view of things, it would be mere tautology to follow up Mr. D's string of questions by as many answers.

As to the self-applauding note, on mine of the 25th, I can only say, that either my diction is very occult, or Mr. D's comprehension very dull. Here I cannot but take to myself his compliment of being a comparatively old practitioner, and lament the haste of youth, so ready to predict good and evil. What was to become of the contained fluid?" Flow out at the urethra, to be sure, and not overcome the sphincter vesicæ. All Mr. D's questions are soluble in the book of experience; his doubts do not appear to arise, like mine, from native dullness and sceptic insufficiency, insufficiency, but because time and study have not matured his perspicuous mind!

I have said, or meant to say, that though, as I wished, the abscess should break internally, that is, into the urethra, yet then, by ulceration, a corresponding opening might be made through the coats of the urethra, and, after all, produce fistula in perinæo; yet better to have this chance, (it may or may not) than to have an internal wound at first: well knowing, that then, from the depth and extent of the abscess, that the sides of the urethra would be destroyed. I would strongly recommend Mr. D. to have more serious ideas of fistula in perinæo, and ask him, how many cases of it he has seen, and had under his care? Pray, is it time to open an abscess when it begins to fluctuate? And must a lancet be employed? Is there no latitude for discretion? No advantages by a degree of spontaneous opening, or the gradual power of poultices? Are there not degrees of suppuration? Mr. D. answers for me, that the process was complete. This implies his idea of full maturation being necessary; but he has made an assertion to which I do not bow; nor to that of *ulceration* producing an opening, &c. The word is not to be found so used by me; no, it was the opening of a large eschar, the apex of the tumour at once disorganized, &c. I may have rendered myself obscure by being too minute; this is not from a cacoethes but a modus scribendi. Had I consulted my vanity instead of my desire in the search of truth, I might possibly have rendered myself intelligible.

Mr. D. had better read again, and see why bougie could not be passed, and consider the danger of passing it while there was so much pain, and the prudence of waiting till the parts were relaxed by bursting of the abscess. As to venereal virus, and coats of the urethra, we are at variance. I shall merely refer Mr. D. to the strict derivative meaning of the words, and also beg him to remember, that the question is yet undecided, which has been so amply discussed by Bell and Hunter. I am accused of want of prudence for endeavouring to preserve the canal and restore its proper diameter, and pave the way to cure by ascertaining the state of the parts; when, only in the preceding paragraph, it is considered singular not to have done so while inflammation existed!

That I did not mean syphilis, must be well known; for, in the outset, it is imputed to an old gonorrhœa. By this Mr. D. may see how I consider the question of lues and gonorrhœa; but they are both venereal affections. He will will now ask, why I speak of virus? Pray, might there not be some syphilitic poison remaining in the patient? And in all wounds, is it not an essential duty to inform ourselves of the constitution, and to be aware of the vices and morbid impressions in the systems of our sufferers?— Let Mr. D. go to his Lexicon for the word urethra, and Charles Bell for an anatomical account of what forms the parietes of that canal; or to any other author he pleases. I imply the whole corpus spongiosum urethræ, which is lined by a continuation of the villous coat of the bladder. Is mercury never used but in syphilitic complaints? What are not its alterative powers? And was the patient totally free from lues? As to the last question, it may be directed to a novice; and for the sake of truth it should be answered, were it possible. But as my head was clear, so my hand was steady; and the aperture, in my impartial eye, was that of ulceration, of which Mr. D. shall have ocular demonstration.

As to consolation, it rests on this; the unceasing confidence of the patient, and the conviction that it was not misplaced; a lasting sense of having done my duty to the extent of my zeal and abilities; which failing to obtain a successful issue for him, have prompted me thus to seek for more knowledge, as a duty to myself, that I may be more extensively enabled to do it to others. The sorrow is, that we cannot command success, although we may do much in deserving it. Few men dare to publish unfavourable terminations. The cause of truth and humanity would flourish much faster, would men do this important duty; in which I mean to proceed on some future day. In the sense of defending myself, and doing justice to my opponents, I shall take final leave of them on this subject, by adding a few quotations.

"In cases of inflammation there is *redness*, tumor, and increased action of the vessels, either of the inflamed part alone, or of the whole system; tension, pain, greater irritability, and an impaired action of the organ affected."

"In cases of inflammation, accompanied with *inflammatory diathesis*, repeated and alternate chilliness, frequently attended with severe rigors, are perceived."

"In the first stage of inflammation, the cure should be attempted by promoting *resolution*, which is effected by (vide the book) and by fomentation, &c.

"Resolution is frequently promoted by blisters, rubefacients, and *other* means of exciting greater action on the vessels in the neighbourhood of the inflamed part."

And under the head of Venereal Disease, "Gonorrhœa may be considered and treated as a local disease, &c."—Vide Elements of Practice of Physic, as delivered by Drs. Saunders and Babington, at Guy's Hospital.*

* Dr. Bellamy will observe, by the corrections we have been compelled to make, that his communications should be previously *revised* by himself. The Editors are well disposed to correct communications; but their emendations, when extensive, are not *always* well received.

